# Renal Imaging Characteristics Predicting Percutaneous Access for Staghorn Stone Management

**DOI:** 10.1007/s11934-026-01365-w

**Published:** 2026-09-18

**Authors:** Vicente Elorrieta, Bahri Efe Turgut, Feres Camargo Maluf, Joseph J. Crivelli, Kyle D. Wood, Thomas Chi, Kazumi Taguchi

**Affiliations:** 1 Department of Urology, The University of Alabama at Birmingham, LHFOT 1123, 510 20th Street S, Birmingham, AL 35294 USA; 2 Ankara University School of Medicine, Ankara, Türkiye

**Keywords:** Staghorn calculi, Percutaneous nephrolithotomy (PCNL), Renal access, Computed tomography (CT), Surgical planning

## Abstract

**Purpose of Review:**

Preoperative planning in staghorn stones treated with percutaneous nephrolithotomy (PCNL) can improve efficiency and minimize complications. We summarized the current evidence on imaging features to guide the selection of optimal access for managing staghorn stones.

**Recent Findings:**

Although the upper pole may offer the most favorable route for stone clearance, it may carry a higher risk of complications in certain situations. In such cases, the middle-calyx may be a safer and equally efficient alternative. Multiple access may still be needed in certain cases. Care should be taken when analyzing preoperative imaging characteristics, as patient positioning and measurements may not directly correlate with the intraoperative findings.

**Summary:**

Detailed assessment of preoperative imaging remains essential for access planning and success. Linear stone burden, distribution of the stone and its relationships to the access calyx, nephrolithometry S.T.O.N.E. score, and infundibular width are relevant factors when planning PCNL access.

**Supplementary Information:**

The online version contains supplementary material available at https://doi.org/10.1007/s11934-026-01365-w.

## Introduction

Staghorn stones represent a complex subgroup of urinary stones that cast the entirety or significant portions of the renal collecting system [1]. Due to the significant comorbidity associated with this condition, management has traditionally been surgical, aiming for aggressive stone clearance [1–3].

According to guidelines, percutaneous nephrolithotomy (PCNL) is the first-line treatment for most patients with complex stones or stones larger than 2 cm [4–6]. Despite advances in urology, treating staghorn stones with PCNL remains challenging. Beyond technical complexity, staghorn cases are associated with higher rates of postoperative complications and longer hospital stays, as well as lower stone-free rates. The PCNL Global Study reported that patients with staghorn stones had a higher rate of multiple punctures (16.9% vs. 5.0%) and a lower stone-free rate (56.9% vs. 82.5%) than non-staghorn undergoing PCNL [7, 8].

To improve outcomes, optimal renal access is paramount as it can facilitate stone clearance while shortening operative time and minimizing complications. Consequently, preoperative imaging plays an essential role in guiding access planning. It can provide detailed information regarding stone distribution, stone size, collecting system anatomy, renal vascularization, and surrounding structures, facilitating individualized PCNL access planning [9]. In this review, we aimed to summarize the current evidence regarding renal imaging features that can assist in selecting optimal access strategies for the management of staghorn stones.

## Methods

A systematic review with narrative synthesis was conducted in May 2026, comprising papers from five databases: PubMed, Embase, Scopus, Web of Science, and Google Scholar (via Publish or Perish). Pre-defined concepts used for data collection and their combination are outlined in Table 1. Due to the intrinsic differences from each search engine, the final search query differed across them (See Supplementary Appendix 1). Terms that did not affect the number of extracted papers were excluded. We also included studies identified from the reference list of screened papers.

**Table 1:** Pre-defined Concepts Applied for the Search Strategy

Concept	Text Word	Controlled Vocabulary
#1 - Patient Population: Patients with staghorn calculi undergoing PCNL	(PCNL OR “percutaneous nephrolithotomy” OR “percutaneous kidney stone removal” OR “minimally invasive nephrolithotomy” OR “percutaneous nephrostolithotomy”) AND (“staghorn calculi” OR “staghorn stone*” OR “complex renal stone*” OR “complete stag*” or “partial stag*”)	“Nephrolithotomy, Percutaneous“[Mesh]; “Staghorn Calculi” [Mesh]
#2 – Exposure: Imaging features (CT, USG, Urography)	(CT OR “computed tomography” OR “3D reconstruction” OR imaging OR ultrasound OR fluoroscopy OR USG OR “CT Urography” OR Urography)	“Tomography, X-Ray Computed“[Mesh]
#3 – Outcome: Optimal Access Planning	(“renal access” OR “access tract*” OR puncture OR “calyceal access” OR “surgical planning” OR “skin-to-stone distance” OR “stone burden” OR nephrolithometry or “preoperative planning” OR “access planning” OR “tract dilation” or “renal puncture”)	
Search Strategy	#1 AND #2 AND #3	

Original studies that evaluated imaging features to plan optimal tract access in patients undergoing PCNL for staghorn stones were included in our review. Papers not written in English, abstracts, systematic reviews, reviews, editorials, guidelines, and opinion letters were excluded. To prioritize the most recent evidence, we applied a primary cutoff of 10 years in the search queries. Despite that, previous studies identified from the reference section of screened papers were included when deemed paramount to address the review question. Details for eligibility criteria, according to the screening phase, are described in Supplementary Appendix 2.

Papers extracted from the databases were inserted in Covidence^®^ for the screening portion. After exclusion of duplicate studies, papers were filtered twice. For the first phase, only the title and abstract were assessed. Then, we evaluated the full text from the filtered ones, outlining the reason for exclusion criteria when needed. This task was conducted by three authors (V.E., E.T., F.C.M). Two authors independently conducted the screening task, and disagreements were resolved by the third author.

From the final pool of papers, we synthesized them in a table format, covering the name of the first author, year of publication, country of research, study design, number of patients, imaging modality, and primary study objective. A more in-depth review was conducted to collect detailed data in subsections considered relevant for access planning: number of tracts, optimal calyx selection, anatomical access point within the calyx, and tract length and size. Ethical approval was not required due to the study design.

## Results

**Fig. 1:**
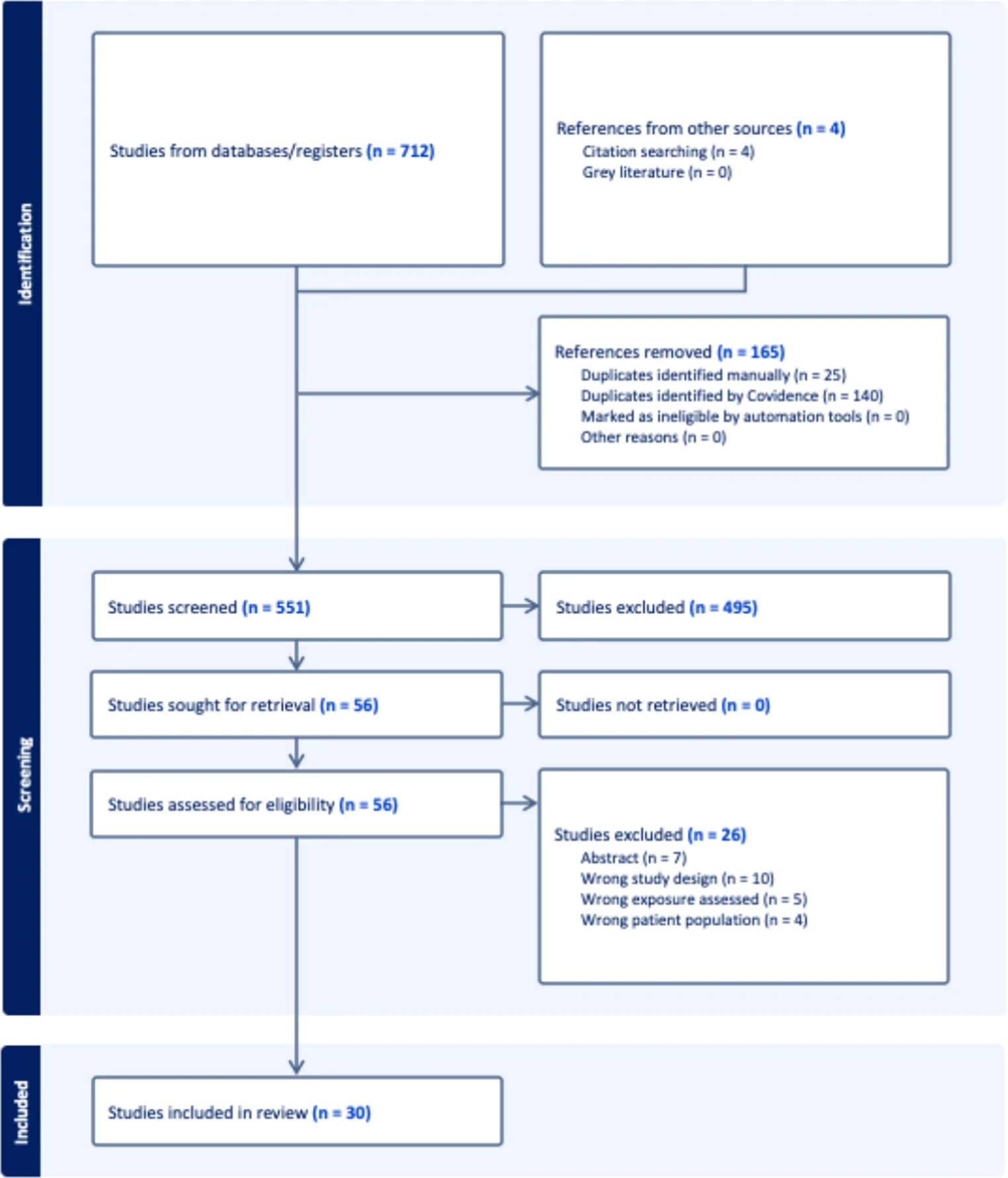
PRISMA Flow Diagram of Study Selection. Flow diagram illustrating the identification, screening, exclusion justifications, and study inclusion. The figure was generated using Covidence

The search strategy yielded 716 papers, out of which 551 were screened after duplicate removal. After the two-stage screening phase, 30 papers were included in the review (Fig. 1). A summary of the included papers is presented in Table 2.

**Table 2:** Overview of Selected Papers

First Author	Year	Country	Design	*N*	Imaging Modality	Primary Study Objective
Manav A	2026	Turkey	Retrospective Cohort Study	181	KUB^a^ X-ray	To evaluate the impact of costovertebral angle width on PCNL^b^ outcomes
Savin Z	2026	USA	Retrospective Study	250	–	To evaluate preoperative predictors of multi-access ECIRS^c^ and ultrasound-guided PCNL
Savin Z	2026	USA	Prospective Study	33	CT^d^ scan	To compare intraoperative tract length with preoperative CT-based estimations using two distinct techniques
Gao L	2025	China	Retrospective Study	323	CT Scan	To evaluate associations between S.T.O.N.E. nephrolithiasis scoring system and PCNL access tract size
Liu Y	2025	China	Pilot	15	CT scan	To evaluate the application of mixed reality technology in the planning of ultrasound-guided PCNL in complex cases
Van der Jeugt J	2025	Spain, Belgium	Retrospective Study	100	Ultrasound, Fluoroscopy	To evaluate the feasibility and safety of upper calyx access in supine PCNL
Wang T	2025	China	Computational Modelling Study	–	Ultrasound	To propose a puncture path planning algorithm for PCNL
Zhang J	2024	China	Retrospective Cohort Study	64	CT Scan	To evaluate the safety and efficacy of 3D^e^ printed models integrated with the Sampaio classification for PCNL access planning
Nayyar R	2023	India	Retrospective Study	136	IV^f^ pyelogram, CT scan	To propose the concept of the ideal “puncture zone” for PCNL and to evaluate its outcomes
Zhu XS	2023	China	RCT^g^	140	CT Scan/Ultrasound	To evaluate the application of ultrasound and CT image overlap in PCNL
Ahn JK	2022	South Korea	Retrospective Cohort Study	69	Ultrasound	To evaluate the feasibility and safety of percutaneous nephrostomy in complex stone cases through two different tract access approaches: behind the stone and renal calyx dilation
Durutovic O	2022	Serbia, UK, Spain, Greece	Pilot	5	CT Scan	To evaluate the impact of CT-based 3D planning software on access acquisition during PCNL.
LaBella D	2022	USA	Retrospective Study	73	CT Scan	To evaluate the utility of performing prone CT scan in preoperative access planning for prone PCNL
Liu Y	2022	China	RCT	72	CT scan	To evaluate the clinical applicability of 3D printing technology for PCNL access planning in staghorn cases
Masarwe I	2022	Israel	Retrospective Study	30	CT scan	To evaluate the reliability of CT for PCNL access planning via exploring the variability of anatomic relations between supine and prone CT
Chow AK	2021	USA	Case-Control Study	43	CT Scan	To evaluate CT scan predictors of thoracic complications in patients undergoing supracostal PCNL
Gupta P	2021	India	RCT	122	Fluoroscopy	To evaluate the clinical value of using air over contrast for retrograde pyelography in PCNL
Huang YS	2021	China	Prospective Trial	120	CT Scan	To evaluate the clinical value of simulated puncture via a CT-based 3D model for PCNL in complex cases
Tan H	2021	China	Retrospective Cohort Study	139	CT Scan	To evaluate the clinical value of CT-based 3D reconstruction model for PCNL
Biswas K	2020	India	Prospective Study	252	CT Scan	To compare the prediction capability of three stone scoring systems for stone-free rates after PCNL
Barut O	2019	Turkey	Retrospective Cohort Study	72	Fluoroscopy	To compare the efficacy and safety of monoplanar versus biplanar access techniques in PCNL
Vande Lune P	2019	USA	Retrospective Study	591	Fluoroscopy	To report a singe-institution experience on fluoroscopy-guided PCNL
Xiao B	2018	China	Case-Control Study/Letter	320	CT Scan	To investigate associations between anatomic parameters of the collecting system and postoperative residual stones in PCNL staghorn patients
Kallidonis P	2017	Greece, Austria	Prospective Study	40	SPECT^h^/CT & CTP^i^	To evaluate the anatomic relations of the papillary, infundibular, and pelvic approaches as well as the amount of vascularization in the respective approaches
Kallidonis P	2017	Greece	RCT	55	Fluoroscopy	To evaluate the safety of infundibular versus papillary access for fluoroscopy-guided PCNL
Basiri A	2016	Iran	Retrospective Study	52	Ultrasound	To evaluate the feasibility of ultrasound-guided PCNL through a proper entry site angled towards the long axis of the target calyx into the renal pelvis
Sofer M	2016	Israel/Italy	Prospective Cohort Study	45	CT scan	To evaluate upper pole accessibility through a lower pole tract access in both prone and supine PCNL
Tailly TO	2016	Canada, Belgium, USA	Retrospective Study	586	CT Scan	To compare three distinct stone scoring systems regarding stone-free rates and postoperative outcomes following PCNL
Verma A	2016	India	Retrospective Study	110	IV pyelogram	To evaluate the impact of pelvicalyceal anatomy, stone morphometry, and site of puncture on PCNL outcomes for complex cases
Mishra S	2012	India	Retrospective Case-control Study	94*	CT scan	To develop a CT urography staghorn morphometry-based prediction algorithm to predict tract number and PCNL stages to obtain stone-free status

*Renal Units; ^a^KUB = kidneys, ureters, and bladder; ^b^PCNL = percutaneous nephrolithotomy; ^c^ECIRS = endoscopic combined intrarenal surgery; ^d^CT = computed tomography; ^e^3D = three-dimensional; ^f^IV = intravenous; ^g^RCT = randomized clinical trial; ^h^SPECT = single-photon emission computed tomography/computed tomography ; ^i^CTP = computed tomography perfusion

Findings were organized into topics considered relevant for evaluation during preoperative planning, using imaging to predict the optimal access for percutaneous nephrolithotomy in patients with staghorn stones.

### Tract Number

This topic was first evaluated by Mishra et al. using CT-based software. The group analyzed the relative stone volume percentage in the pelvicalyceal system, dividing it into the calyx of entry, the pelvis, and the remaining calyces. For the latter, it was further categorized by accessibility. Favorable calyces were defined as those with an obtuse angle relative to the entry calyx and an infundibulum width greater than 8 mm. They found that patients were more likely to require multiple tracts when a greater proportion of the total stone burden was located within unfavorable calyces [10]. In contrast, a greater percentage of the total stone burden located in the calyx of entry and the pelvis was associated with single-tract PCNL. They also proposed a model in which a lower total stone volume with limited unfavorable calyceal involvement was more likely to be managed with a single-tract, single-stage PCNL procedure.

Other studies have also suggested that the number of access tracts is closely related to stone complexity and collecting-system anatomy. In a retrospective study of 320 patients, Xiao et al. found that among patients undergoing PCNL for staghorn stones, residual-stone cases required more tracts and had significantly narrower infundibular width (6.0 ± 1.7 mm vs. 11.2 ± 1.4 mm, *p* < 0.001), whereas infundibular length, infundibular-pelvic angle, and upper-lower calyceal angle were not predictive. They concluded that narrow infundibular anatomy of the punctured calyx may restrict rigid nephroscope maneuverability and increase the likelihood of requiring additional access [11]. Biswas et al. similarly found that patients with residual stones had a higher mean number of tracts than stone-free patients in a prospective PCNL cohort [12].

Verma et al. studied 110 patients undergoing standard PCNL and examined how pelvic and calyceal anatomy influenced the need for multiple access tracts. They measured the pelvicalyceal system surface area using a 1-mm^2^ grid from IVU, the infundibular width at the narrowest point of the entry calyx, and the intercalyceal angle defined as the angle between the central axis of the entry calyceal infundibulum and the nearest calyx. Patients were grouped into those who achieved stone clearance with a single puncture (Group 1) and those requiring more than one puncture (Group 2). A smaller pelvicalyceal surface area (134.27 mm^2^ vs. 109.9 mm^2^, *p* = 0.01), a narrower infundibular width (5.9 mm vs. 4.41 mm, *p* < 0.001), and a more acute intercalyceal angle (78.49° vs. 63.42°, *p* = 0.01) were predictors of the need for multiple accesses. Additionally, stone distribution across all three calyces, particularly in the middle and lower calyces, was associated with the need for additional punctures, highlighting these factors as unfavorable for successful single-tract PCNL [13].

Savin et al. recently examined predictors of multi-access in a cohort of 250 patients undergoing US-guided supine PCNL. The initial access point in the cohort was primarily the lower pole (59% of cases). They found that a linear stone burden greater than 34.5 mm was associated with a higher likelihood of requiring multiple accesses. Interestingly, contrary to previous literature, their data showed that in patients with non-staghorn stones, upper-pole involvement was a strong predictor of the need for multiple tracts. Nevertheless, their results should be interpreted carefully, given that the initial access was primarily through the lower pole. The authors suggest that avoiding initial upper pole access could lead to increased use of multiple tracts, indicating that these patients might have been better approached with an initial non-lower-pole access.

### Optimal Calyx Selection

Theoretically, in staghorn stones, the upper calyx offers smoother access to the ureteropelvic junction, providing wider access to other calyces and reducing the need for ancillary tracts [14]. However, superior calyx access may be associated with higher risks of pleural complications and adjacent organ injuries (e.g., liver or spleen). Vande Lune et al. reported retrospective data on 591 patients treated with PCNL and found a significantly lower need for additional tracts with upper pole access compared with middle/lower pole access (13.7% vs. 33.3%, *p* = 0.02) [15]. On the other hand, they found a higher incidence of major complications with upper pole access (10.1% vs. 3.6%, *p* = 0.008), such as pleural effusions requiring treatment (5.4%), post-procedural venous thromboembolism (2.7%), and renal pseudoaneurysm requiring treatment (1.6%). More specifically, a supracostal vs. infracostal approach to the upper pole was associated with higher rates of both overall (34.1% vs. 18.7%; *p* = 0.002) and major complications (12% vs. 4.7%, *p* = 0.04). For supracostal punctures, the most common complication was hemothorax or hydrothorax requiring chest tube placement (2.9%). In the infracostal puncture group, the most common major complication was renal pseudoaneurysm requiring embolization (1.9%).

On a closer evaluation of the supracostal approach, Chow and colleagues evaluated preoperative CT scans in the patients undergoing PCNL with supracostal access to determine the likelihood of thoracic complications. While the relationship of the upper renal pole to the 12th rib and the costophrenic angle was not predictive, a diaphragmatic insertion ≤ 2.5 cm below the upper renal pole was predictive of thoracic complications [16]. On the other hand, Van der Jeugt et al. found that upper calyx access in supine PCNL can be feasible and safe, reporting an 85% single-procedure stone-free rate without intraoperative complications or significant thoracic, hepatic, or splenic injuries, despite frequent supracostal access [17].

Despite the recognized advantages of upper pole access, recent studies have evaluated the safety and success rates of middle-calyx puncture in staghorn stones. Verma et al. described in their series of 110 patients that a middle-calyx puncture yielded similar results with upper-calyx puncture in terms of stone clearance rates and complications [13]. In a recent study by Manav et al., narrow costovertebral angle (CVA) values (< 45 degrees) were evaluated as a potential cause of higher complications in patients undergoing middle-calyx access, though no statistically significant association was found [18]. Conversely, a lower pole access may be preferable in patients with a stone distribution limited to the renal pelvis and/or a single calyx in the lower pole, with no involvement of parallel calyces [10, 19].

### Anatomical Access Point Within the Calyx

In a study by Kallidonis et al., the safety of papillary, infundibular, and pelvic punctures to the middle renal calyx was evaluated in 40 patients using SPECT/CT and CT perfusion. They found no significant differences in vascularization or angle of approach among the three approaches [20].

The same authors also conducted a randomized clinical trial (RCT) comparing 27 patients in the papillary puncture group and 28 patients in the infundibular (non-papillary) puncture group during PCNL, testing the paradigm that papillary access is safer. Overall complication rates did not differ between groups (7.4% vs. 7.14%). There was no significant difference in hemoglobin loss (1.54 g/L vs. 1.35 g/L, *p* = 0.92), creatinine change (-0.04 mg/dL vs. 0.04 mg/dL, *p* = 0.89), or hospital stay in days (5.57 vs. 5.8, *p* = 0.7) between groups. In addition, papillary access was associated with a longer operative time (51.97 ± 16.1 vs. 43.21 ± 12.38 min, *p* = 0.027) [21]. They also noted as a limitation that this small-sample study lacked treatment outcome data, including stone-free rate, and long-term follow-up data.

In searching for an ideal puncture site for each calyx, Nayyar et al. approached tract selection from an anatomical and geometric perspective and introduced the concept of a three-dimensional “puncture zone” for each calyx. In this model, each calyx is conceptualized as a cone, and tracts are considered favorable if they enter through the base of this cone toward the calyx [21].

Kallidonis et al. investigated papillary, infundibular, and pelvic approaches to the middle calyx using SPECT/CT and CT perfusion imaging and found no significant differences in angle of approach or regional parenchymal vascularity among these access routes, suggesting that selected non-papillary trajectories may be anatomically comparable to conventional papillary access in appropriately selected cases [22].

### Tract Length and Size

When discussing the optimal access tract for PCNL, the literature has evaluated parameters such as tract length and size. LaBella et al. demonstrated that tract length measured on supine CT may change after prone repositioning, suggesting that prone CT may be particularly useful when an exceptionally long tract length (> 15 cm) is anticipated [23]. In a similar study, Savin et al. showed that CT-based tract length measurements significantly overestimated real-time intraoperative tract length during supine PCNL, with a mean intraoperative length of 9.0 ± 2.5 cm compared with 11.2 ± 2.7 cm using a CT-anatomical method and 11.4 ± 2.2 cm using the S.T.O.N.E.-based method [24].

Notably, when ultrasound-guided access is performed by experienced operators, adjacent organs and structures to avoid during puncture can be assessed in real time, enabling safer access regardless of the selected calyx. The puncture site and trajectory are determined by the shortest skin-to-calyx distance to achieve the most direct and safest access path [25]. The ideal needle angle has been described as the one that aims at the pelvis and aligns with the long axis of the target calyx [26, 27].

Selection of tract size is another important component of PCNL planning. Guidelines recommend standard PCNL (24 Fr to 30 Fr) for the treatment of complete staghorn stones and stones larger than 3 cm [4, 28]. However, mini-PCNL techniques (less than 22 Fr) have gained popularity over the past two decades because of their lower morbidity profile and potential advantages in anatomically challenging situations, such as impacted lower-pole stones with acute infundibular angles, calyceal diverticular stones, and difficult-to-access stone locations. Furthermore, the incorporation of newer technologies, including suction-enabled access sheaths and high-power laser systems, has expanded the applicability of mini-PCNL, allowing stone-free rates comparable to those achieved with standard tracts in selected patients. For stones up to 3 cm, standard and mini-PCNL provide similar stone-free rates. However, there are ongoing concerns about longer operative times and lower efficiency when dealing with larger or more complex stones. Multiple studies have shown that for stones near or exceeding 3 cm, standard PCNL tends to achieve higher stone-free rates [29–31].

To clarify the role of tract size in relation to stone complexity, Gao et al. retrospectively compared 149 patients who underwent standard-tract PCNL (SPCNL) with 174 who underwent mini-PCNL (MPCNL), stratifying cases by the S.T.O.N.E. nephrolithometry score into low-complexity (5–8 points) and high-complexity (9–13 points) groups. Among patients with high-complexity scores, standard-tract was associated with higher stone-free rates (72.6% vs. 33.3%, *p* < 0.001) and shorter operative times than MPCNL (112.5 vs. 134.0 min, *p* < 0.001), while complication rates remained similar. On multivariable analysis, SPCNL was an independent protective factor against residual fragments (OR 2.88, 95% CI 1.52–5.48) [32].

### Additional Technical Considerations

Patient positioning is among the factors that influence calyx selection in patients undergoing PCNL. In the prone position, the nephrostomy tract length is shorter. In contrast, in the supine position, kidneys situated deeper in the abdomen have a wider access angle [25]. Masarwe et al. compared supine with prone positioning during preoperative CT, identifying the kidney demonstrated posterior rotation, slight lateral movement, and cranial displacement during the transition from prone to supine, with left-sided upward movement potentially bringing the upper pole closer to the diaphragm. The authors suggest that patients’ positions should be the same during CT imaging and PCNL [33]. In another study evaluating patient positioning during CT, prone positioning was associated with a higher risk of organ interceptions.

Gupta et al. investigated intraoperative imaging adjuncts for fluoroscopy-guided renal access by comparing retrograde pyelography with contrast, air, and a combination of both during PCNL. The authors hypothesized that posterior calyceal access, which is generally preferred in prone PCNL due to its favorable trajectory and reduced risk of vascular injury, may be hindered by conventional contrast agents, as they tend to accumulate in anterior calyces and may inadequately opacify posterior calyces. Conversely, air preferentially rises into the posterior calyces, facilitating their identification and access. In this interim analysis of a RCT, 50 patients underwent contrast-assisted puncture, 53 underwent air-assisted puncture, and 19 underwent puncture using both air and contrast. Compared with the contrast group, patients undergoing air-assisted puncture experienced lower radiation exposure and fluoroscopy time, shorter access times, and higher stone-free rates [34].

An additional concept related to access planning is needle entry in relation to stone distribution and the presence of hydronephrosis. Although studied in the context of percutaneous nephrostomy rather than PCNL, Ahn et al. retrospectively evaluated 69 patients with renal stones, including partial (51.4%) and complete (12.9%) staghorn calculi, who underwent renal puncture either through a dilated calyx or directly behind the stone. Technical success was achieved in all patients, and complication rates were similar between the two groups (20.0% vs. 25.4%, *p* = 0.279) [35].

Additional imaging methods such as mixed reality and three-dimensional (3D) model, are particularly advantageous in complex stones or anatomically challenging cases [26]. In a 15-patient pilot study, using CT urography data, digital 3D reconstructions were created and projected into space to assist with calyx selection and tract planning. All procedures were completed successfully; the consistency between preoperative planning and intraoperative execution was 87.6%, the stone-free rate was 80%, and no Clavien-Dindo grade ≥ II complications were reported [36]. In another study, 3D models were used as adjunctive tools for preoperative access planning. Patients were divided into two groups: Experimental (3D Models) and Control (CT imaging). The experimental group not only had a significantly shorter mean puncture time (2.1 min vs. 3.5 min, *p* = 0.024), but also higher mean target calyx consistency (88.3 vs. 61.4, *p* = 0.013) and a higher mean stone-free rate (83.1% vs. 69.2%, *p* = 0.014) [37]. Another potential use for 3D modeling is “simulated puncturing,” which refers to creating a 3D reconstruction of the collecting system, then using the model to choose the target calyx and design the best puncture channel. Compared to CT scanning alone, simulated puncture when added to CT was associated with shorter operative time, fewer puncture attempts, fewer access channels, and less intraoperative blood loss [38]. It was further shown that integrating 3D reconstruction/printing with the Sampaio classification improved target calyx consistency, reduced puncture time and the number of puncture channels, and increased stone-free rates in complex renal stones [39]. Similarly, another study reported that overlapping ultrasound and CT images enabled to select an optimal puncture channel, resulting in higher single puncture success rate, fewer number of the access tracts, less postoperative hemoglobin reduction, shorter operative time, and improved first-stage stone clearance [40]. Wang et al. extended this concept computationally by proposing a two-step puncture path planning algorithm that selects the puncture calyx based on projected stone accessibility and then optimizes the puncture path according to length, smoothness, and safety [36].

## Final Considerations

Preoperative planning of the puncture trajectory and tract characteristics is a critical step for successful PCNL. Awareness and understanding of the kidney’s gross, surgical, and radiologic anatomy will help optimize outcomes, prevent potential complications, and, if they occur, enable timely recognition and efficient management [25].

Guidelines recommend obtaining at least a non-contrast CT before surgery in patients undergoing PCNL for kidney and/or ureteral stones [4]. CT facilitates assessment of stone burden, density, and the previously mentioned optimal sites for percutaneous renal access. It also provides important information on the relationship of the kidneys to adjacent organs and on challenging anatomical abnormalities such as renal cysts, renal masses, scars, and calyceal diverticula. CT also provides useful information about skin-to-stone distance and stone volume.

Currently, three major validated characterizing systems are used to predict success rates and the risk of complications in PCNL: Guy’s Stone Score (GSS), the CROES nomogram, and nephrolithometry S.T.O.N.E. score. These tools can help determine the surgical modality and estimate success and complication rates, helping manage expectations for both patients and surgeons. However, they were not originally designed to identify the optimal access route or the number of tracts required [12, 19]. Therefore, a detailed assessment of CT imaging characteristics remains essential for individualized access planning and procedural success. Additionally, multiple adjunctive tools are now available to improve preoperative imaging planning, such as CT with urography, 3D reconstructions, and 3D printing of the vascularization and collecting systems. Considering these tools for selected or complex cases may improve surgical outcomes.

While CT imaging offers clear advantages, clinicians should interpret the images cautiously for preoperative planning. Differences between the planned and actual patient positions during the scan can alter the analysis, influencing the predicted angles and trajectories for needle entry. These variations need careful consideration. Moreover, new research shows that CT-based measurements of tract length tend to considerably overestimate the true intraoperative length during supine PCNL, highlighting the need for thorough evaluation to achieve the best surgical planning.

Due to the inherent complexity and involvement of multiple calyces, multiple tracts may be used to treat staghorn stones [41]. Despite current advances such as endoscopic combined intrarenal surgery (ECIRS), anatomical and technical factors may make single-procedure complete stone clearance unfeasible [42]. Reports indicate that multi-access may be required in up to 7% of PCNL procedures and up to 17% of staghorn stones, depending on the surgeon’s experience [43]. Distribution of the stone burden and linear measurement of the stone may predict the need for multiple access.

Selecting the optimal calyx for access may improve stone-free rates and surgical efficiency. Important considerations to evaluate include stone distribution, kidney anatomy and location, stone-to-skin distance, surrounding fat thickness and nearby organs, hydronephrosis and obstruction and parenchymal thickness [19]. Traditionally, the surgical approach to PCNL access recommends the upper calyx for treating complete staghorn stones [41], but recent research has examined the safety and success rates of middle-calyx puncture, which may be suitable for patients at higher risk of thoracic complications [13]. Conversely, a lower pole access may be preferable in patients with a stone distribution limited to the renal pelvis and/or a single calyx in the lower pole, with no involvement of parallel calyces [10, 19]. Therefore, it appears that a key factor in determining the most efficient initial access is the distribution of the main stone burden within the collecting system.

## Conclusions

CT remains the primary imaging modality for evaluating the optimal access route during preoperative planning for PCNL, though mixed reality and 3D modeling are gaining interest among urologists. Detailed assessment of preoperative imaging remains essential for access planning and success. Relevant factors to consider in the preoperative evaluation of images include linear stone burden, distribution of the stone and its relationship to the access calyx, the nephrolithometry S.T.O.N.E. score, and infundibular width.

## Key References

- Savin Z, Geffner AD, Frangopoulos E, Durbhakula V, Rahmani LD, Mandel A, et al. Who still needs multiple access sites in the era of ECIRS and US-guided supine PCNL? World J Urol. 2026;44(1):105.
  - ○ Perioperative predictors of multi-access PCNL in the era of ECIRS
- Savin Z, Gupta R, Kaphle S, Gupta K, Frangopoulos E, Durbhakula V, et al. Real-Time Tract Length During Supine Percutaneous Nephrolithotomy Is Shorter than on Preoperative Imaging: Implications for Surgical Planning. J Endourol. 2026;40(4):447-54.
  - ○ Investigated concordance between preoperative CT measurement of tract length and intraoperative measurement
- Wang T, Nie Z, Xu Y, Su B, Xie F, Wang J, et al. Puncture path planning algorithm of flexible needle in percutaneous nephrolithotomy with a hybrid NSGA optimizer. Comput Methods Programs Biomed. 2025;266:108763.
  - ○ Evaluation of an algorithm for preoperative PCNL puncture-path planning

## Supplementary Information

Below is the link to the electronic supplementary material.

Supplementary Material 1

Supplementary Material 2

## Data Availability

All data supporting the findings of this study are available within the paper and its Supplementary Information.
