# Supplementary material for: Renal Imaging Characteristics Predicting Percutaneous Access for Staghorn Stone Management
Source: Curr Urol Rep. 2026 Sep 18;27(1):49. doi: 10.1007/s11934-026-01365-w (PMC13588915; doi:10.1007/s11934-026-01365-w)
Supplement: Supplementary file 1 [file 11934_2026_1365_MOESM1_ESM.docx]

**Supplementary Appendix 1**

**Search Queries**

PubMed (05/01/2026)

Query: ("Nephrolithotomy, Percutaneous"[Mesh] OR PCNL OR "percutaneous nephrolithotomy" OR "percutaneous kidney stone removal" OR "minimally invasive nephrolithotomy" OR "percutaneous nephrostolithotomy") AND ("Tomography, X-Ray Computed"[Mesh] OR CT OR "computed tomography" OR "3D reconstruction" OR imaging OR ultrasound OR fluoroscopy OR USG OR "CT urography" OR urography) AND ("renal access" OR "access tract*" OR puncture OR access OR "calyceal access" OR "surgical planning" OR "skin-to-stone distance" OR "preoperative planning" OR "access planning" OR "tract dilation" OR "renal puncture") AND ("Staghorn Calculi" [Mesh] OR "staghorn calculi" OR "staghorn stone*" OR "complex renal stone*" OR "complete stag*" OR "partial stag*" OR staghorn) AND (y_10[Filter])

Embase (05/01/2026)

Query: ('percutaneous nephrolithotomy'/exp OR 'percutaneous nephrolithotomy') AND ('imaging'/exp OR imaging) AND ('staghorn stone'/exp OR 'staghorn stone') AND [2016-2026]/py:

Scopus (05/01/2026)

Query: ( ALL ( pcnl percutaneous nephrolithotomy ) AND ALL ( imaging ) AND ALL ( staghorn stone ) AND ALL ( tract access ) ) AND PUBYEAR > 2015 AND PUBYEAR < 2027: 228 findings

Web of Science (05/01/2026)

Query: ((((TS=(pcnl)) AND TS=(imaging)) AND TS=(staghorn)) AND TS=(access)) AND PY=(2016-2026): 42 findings

Google Scholar (05/01/2026)

Query: ("percutaneous nephrolithotomy"|PCNL|"percutaneous kidney stone removal"|"minimally invasive nephrolithotomy"|"percutaneous nephrostolithotomy") ("computed tomography"|CT|"3D reconstruction"|ultrasound|fluoroscopy|imaging|"CT urography"|urography) ("renal access"|"access tract"|puncture|"calyceal access"|"surgical planning"|"skin to stone distance"|"preoperative planning"|"access planning"|"tract dilation"|"renal puncture") ("staghorn calculi"|"staghorn stone"|"complex renal stone"|"complete staghorn"|"partial staghorn"|staghorn). Limit set to 200 results
