# Supplementary material for: Renal Imaging Characteristics Predicting Percutaneous Access for Staghorn Stone Management
Source: Curr Urol Rep. 2026 Sep 18;27(1):49. doi: 10.1007/s11934-026-01365-w (PMC13588915; doi:10.1007/s11934-026-01365-w)
Supplement: Supplementary file 2 [file 11934_2026_1365_MOESM2_ESM.docx]

Supplementary Appendix 2

**Supplementary Table 1. Inclusion and Exclusion Criteria Applied for the Article Screening Stage**

| **Screening Stage** | **Include** | **Exclude** |
| --- | --- | --- |
| Title/Abstract Level | - Studies discussing PCNL access strategies for staghorn stones through imaging parameters - Studies including PCNL in staghorn patients; - Studies mentioning imaging for PCNL access planning; | - Not in English; - Abstracts; - Systematic reviews, reviews, guidelines, editorials, and opinion letters; - No mention of PCNL in staghorn patients; - No mention of imaging for PCNL access planning; |
| Full Article Level | - Studies associating imaging features with access tract planning in staghorn patients undergoing PCNL; | - Wrong study design; - Abstracts - Studies not associating imaging features with access tract planning in staghorn patients undergoing PCNL; - Studies only assessing non-staghorn stones; |
